# Merger of dynamic two-photon and phosphorescence lifetime microscopy reveals dependence of lymphocyte motility on oxygen in solid and hematological tumors

**DOI:** 10.1186/s40425-019-0543-y

**Published:** 2019-03-18

**Authors:** Mateusz Rytelewski, Karine Haryutyunan, Felix Nwajei, Meenakshi Shanmugasundaram, Patrick Wspanialy, M. Anna Zal, Chao-Hsien Chen, Mirna El Khatib, Shane Plunkett, Sergei A. Vinogradov, Marina Konopleva, Tomasz Zal

**Affiliations:** 1 Department of Immunology, University of Texas MD Anderson Cancer Center, U902, 7455 Fannin St, Houston, 77054 TX USA; 2 Department of Leukemia, University of Texas MD Anderson Cancer Center, Houston, TX USA; 3 School of Engineering, University of Guelph, Guelph, Canada; 4 Departments of Biochemistry and Biophysics and of Chemistry, University of Pennsylvania, Philadelphia, PA USA

**Keywords:** Tumor microenvironment, Tumor infiltrating lymphocytes, T cell motility, Tissue oxygenation, Intravital microscopy, Acute lymphocytic leukemia, Lung sarcoma tumors, Two-photon lifetime imaging, PLIM

## Abstract

**Background:**

Low availability of oxygen in tumors contributes to the hostility of the tumor microenvironment toward the immune system. However, the dynamic relationship between local oxygen levels and the immune surveillance of tumors by tumor infiltrating T-lymphocytes (TIL) remains unclear. This situation reflects a methodological difficulty in visualizing oxygen gradients in living tissue in a manner that is suitable for spatiotemporal quantification and contextual correlation with individual cell dynamics tracked by typical fluorescence reporter systems.

**Methods:**

Here, we devise a regimen for intravital oxygen and cell dynamics co-imaging, termed ‘Fast’ Scanning Two-photon Phosphorescence Lifetime Imaging Microscopy (FaST-PLIM). Using FaST-PLIM, we image the cellular motility of T-lymphocytes in relation to the microscopic distribution of oxygen in mouse models of hematological and solid tumors, namely in bone marrow with or without B-cell acute lymphocytic leukemia (ALL), and in lungs with sarcoma tumors.

**Results:**

Both in bone marrow leukemia and solid tumor models, TILs encountered regions of varying oxygen concentrations, including regions of hypoxia (defined as pO_2_ below 5 mmHg), especially in advanced-stage ALL and within solid tumor cores. T cell motility was sustained and weakly correlated with local pO_2_ above 5 mmHg but it was very slow in pO_2_ below this level. In solid tumors, this relationship was reflected in slow migration of TIL in tumor cores compared to that in tumor margins. Remarkably, breathing 100% oxygen alleviated tumor core hypoxia and rapidly invigorated the motility of otherwise stalled tumor core TILs.

**Conclusions:**

This study demonstrates a versatile and highly contextual FaST-PLIM method for phosphorescence lifetime-based oxygen imaging in living animal tumor immunology models. The initial results of this method application to ALL and solid lung tumor models highlight the importance of oxygen supply for the maintenance of intratumoral T cell migration, define a 5 mmHg local oxygen concentration threshold for TIL motility, and demonstrate efficacy of supplementary oxygen breathing in TIL motility enhancement coincident with reduction of tumor hypoxia.

**Electronic supplementary material:**

The online version of this article (10.1186/s40425-019-0543-y) contains supplementary material, which is available to authorized users.

## Background

The capacity of lymphocytes to actively migrate within tissue is critical for the immune response to cancer [1, 2]. In tumors, including bone marrow malignancies [3–8], infiltrating lymphocytes (TIL) encounter a hostile microenvironment that is often depleted of oxygen. Low interstitial oxygen concentrations contribute to tumor immune evasion as strikingly evidenced by the benefit brought about supplementary oxygen breathing during experimental anti-tumor immunotherapy in mice [9]. There are multiple mechanisms by which oxygen availability modulates TIL function, including by promoting tissue expression of checkpoint ligands and by decreasing antigen presentation and TIL cytotoxicity [10, 11]. However, the relationship between oxygen availability and the ability of TILs to carry out migratory surveillance of tumor masses remains unclear.

This situation reflects a general difficulty in the microscopic imaging of living tissue oxygenation together with cell dynamics. Among the available methods, nitroimidazole adduct formation requires ex vivo tissue staining whereas reporters of hypoxia inducible factor pathway and carbonic anhydrase activity are only moderately correlated with oxygen, and hence imprecise and cell-type dependent [12–15]. The most promising method for this purpose is two-photon microscopy based on phosphorescence lifetimes. In this technique, which was greatly improved with the introduction of special two-photon-enhanced phosphorescent probes, such as PtP-C343 [16], partial pressures of oxygen (pO_2_) are determined based on phosphorescence lifetime [17–20], which is unaffected by probe concentration [21]. Thus far, two-photon phosphorescence lifetime microscopy of oxygen has been realized either as high speed measurements in discrete locations in vivo [17, 22, 23], or as imaging by conventional scanning in vitro [16, 24]. Unfortunately, due to long triplet decay times and, consequently, inherently low photon arrival rates, the temporal performance of conventional two-photon phosphorescence lifetime microscopy becomes limiting when this modality is used for imaging (PLIM). Typically, the time needed for acquisition of a high-resolution oxygen image is up to hours, which is one to two orders of magnitude slower than the frame rate required for reliable tracking of fast moving T cells (~ 2 frames per minute). Although, in principle, frame rates in PLIM can be improved using gated cameras [25, 26], or frequency-domain spatial multiplexing [27], these PLIM modes lack depth resolution or are strongly confounded by additional fluorescence. Due to this performance gap between two-photon phosphorescence and classical two-photon microscopy, these powerful modalities are yet to be merged in a practical manner.

Here, we analyze the relationship between lymphocyte motility and oxygen distribution in healthy or leukemic bone marrow, and in solid lung tumors, using bi-modal imaging that merges traditional fluorescence two-photon microscopy with imaging-optimized two-photon microscopy of phosphorescence lifetimes.

## Methods

### Development of contextual intravital imaging of oxygen and cell dynamics

**Fig. 1:**
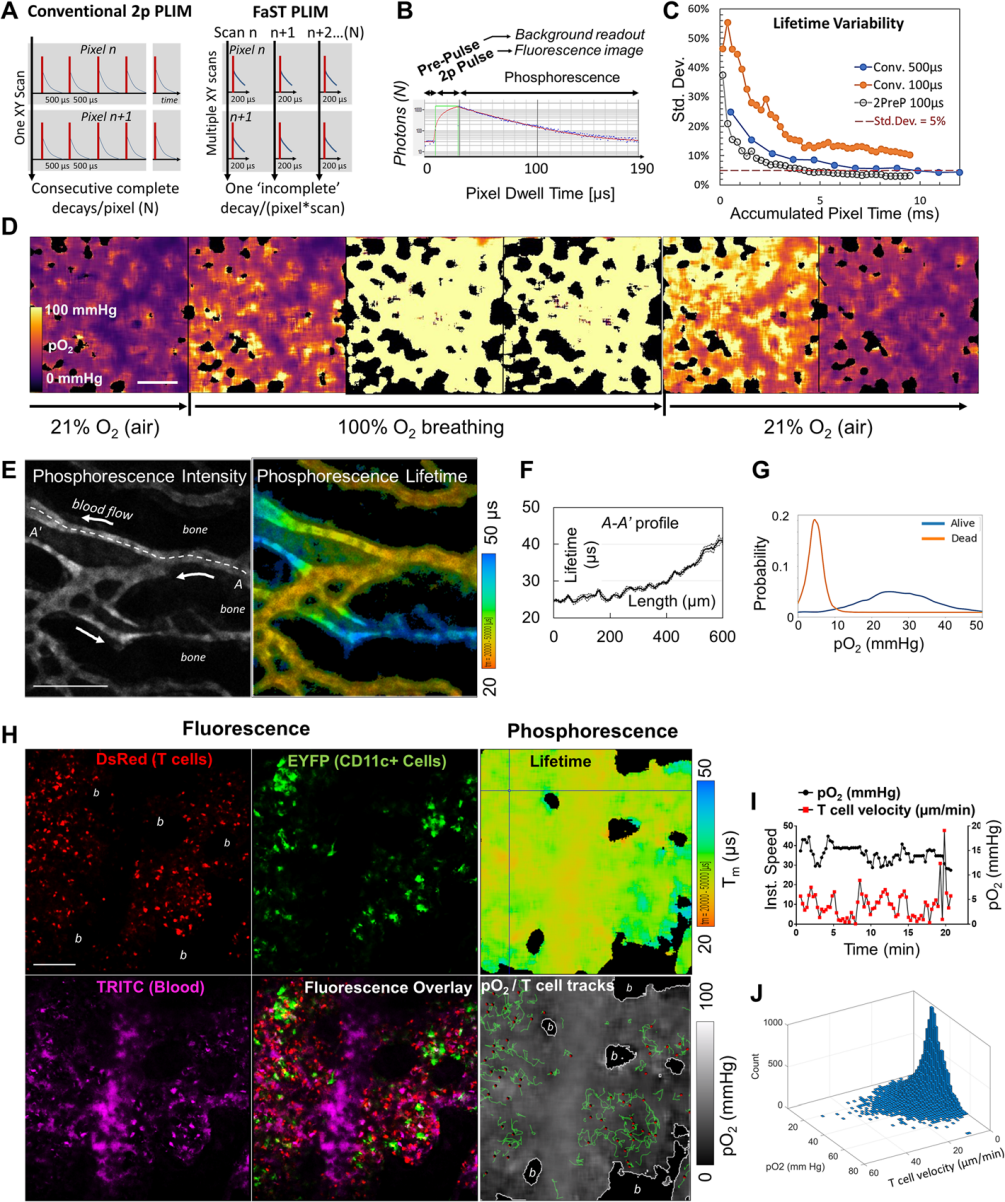
FaST-PLIM method for intravital contextual imaging of oxygen and T cell dynamics. **a** FaST-PLIM scanning design. In conventional two-photon (2p) PLIM, each pixel receives one series of excitation gates and each unitary decay measurement period is at least > 10 lifetimes (complete decay). In FaST-PLIM, each pixel receives only one excitation gate per scan and the unitary decay measurement period is substantially shorter than 10 lifetimes (‘incomplete decays’). **b** Design of individual pixel dwell time whereby the excitation gate (2p Pulse) is preceded by Pre-Pulse. Blue dots represent photon counts, the red line is fitted model. **c** Results of Monte Carlo modeling of the dependence of lifetime variability on the accumulated pixel time. “Conv. 500 μs” and “Conv. 100 μs” represent, respectively, ‘complete’ or partial decay conventional methods, i.e. without pre-pulse, and with the corresponding pixel dwell times. “2PreP 100 μs” represents a partial decay method with pre-pulse and a 100 μs pixel dwell time. Model probe lifetime was 50 μs. **d** Intravital FaST-PLIM of oxygen (PtP-C343 probe) in lung. The mouse was initially ventilated with atmospheric air (~ 21% O_2_) followed by 100% O_2_, and then atmospheric air once more. The black areas correspond to alveolar air spaces and indicate no signal from the probe. **e** Intravital FaST-PLIM of in vivo oxygen in mouse calvarial BM. The arrows indicate blood flow direction. Lifetime image was intensity-weighted. **f** Lifetime values along the *A-A’* dashed line. **g** Spatial frequency distribution of BM pO_2_ before (Alive, orange line) and after specimen asphyxiation (Dead, blue line). **h** Oxygen and fluorescence dynamics in calvarial BM of healthy CD2-DsRed/CD11c-YFP mouse infused with TRITC-dextran (blood supply marker) and PtP-C343. “*b*” indicates bone areas in which no signal from the probe was recorded. **i** Example of pO_2_ experience of an individual T cell as it moves along its track. **j** Overall frequency of instantaneous T cell velocities and local pO_2_

To relate dynamic cellular processes such as motility to the local availability of oxygen in vivo, we needed to develop an imaging method that combines two-photon phosphorescence lifetime microscopy with time-lapse imaging of fluorescence. In general, measurement of phosphorescence lifetime in time domain involves signal accumulation from multiple acquisition cycles whereby each unitary acquisition consists of a short excitation gate followed by recording of phosphorescence photon arrival times. In a typical, spot-wise measurement regimen, all the required unitary acquisition cycles are executed one after another for each measurement position. When applied to imaging, this traditional PLIM regimen results in very long pixel dwell times, which situation is incompatible with dynamic imaging. To overcome this limitation, we designed a ‘fast’ scanning two-photon (FaST) PLIM regimen that differs from the conventional one in the manner of signal accumulation and in the unitary acquisition design. For signal accumulation, we used only one excitation/acquisition cycle per each pixel visit in repeated scanning of the entire field of view, as opposed to the multiplicity of excitations for each pixel dwell time in a singular frame scan in the conventional regimen for time domain PLIM (Fig. 1a). Compared to the conventional regimen, the use of *frame priority* scanning did not shorten the overall duration of lifetime image acquisition, but it attained a much faster fundamental scanning rate i.e., in proportion to the required number of unitary decay acquisitions per pixel (about ten to hundred times faster in our application). Thus, in conjunction with recording fluorescence during each excitation gate, whose duration was appropriately optimized, the frame priority mode of phosphorescence signal accumulation enabled much higher frame rate for concurrent cell dynamics recording than it would be possible conventionally. We implemented this scanning regimen using a commercially available galvanometer scanner with photon counting detectors and dynamic range-extended electro-optical laser gating (Additional file 1: Figure S1).

Nonetheless, although greatly improved, the attained fluorescence scanning rate of about one frame per minute was still too slow for tracking of fast moving cells such as T cells. In the next step of method development, we considered the unitary acquisition cycle duration, which is determined by the temporal spacing of consecutive excitation gates. In the conventional PLIM regimen, the consecutive excitation gates must be spaced by at least ~ 10 decay times to prevent triplet state pile-up [16, 24]. However, because our regimen separates the consecutive excitation gates in space, i.e., between consecutive pixels, the time between individual excitation gates in each pixel is as long as one scan duration (e.g., tens of seconds). Therefore, no longer constrained by triplet state pile up, we could shorten each pixel dwell time by measuring only a fraction of each decay, known as ‘incomplete decays’ (Fig. 1a and b). However, in silico simulations revealed large standard deviations in lifetime determination from incomplete decays when using the conventional three-parametric fitting, i.e. amplitude, lifetime, and offset (background) (Fig. 1c). Therefore, to improve the fit accuracy, we added a short “Pre-Pulse” period prior to each excitation gate, which provided an additional independent measurement that in principle corresponds to the offset (Fig. 1b). With the offset measured by the pre-pulse, the decay could be fitted using a single-exponential model with only two parameters: amplitude and lifetime. In silico simulations showed that standard deviations in lifetime estimations from incomplete decays with pre-pulse were significantly reduced compared to those without pre-pulse, and were similar to those determined using ‘complete’ decays (Fig. 1c and Additional file 1: Figure S2A and B). This way, not only was the scanning rate improved by additional ~ 2.5-fold, but the speed of lifetime image acquisition was improved by ~ 2.5-fold for a chosen level of precision (e.g., 5% error in lifetime estimation), compared to that using the complete decays regimen. Taken together, by combining frame-priority scanning with pre-pulse-enabled incomplete decay measurements in the FaST-PLIM regimen, we could obtain one high resolution lifetime image for PtP-C343 in as little as one to several minutes while imaging fluorescence at the desired rate of 2 images per minute. A more detailed description of the animal models and imaging and in silico modeling procedures is provided in the subsequent sections.

#### Mouse acute lymphocytic leukemia imaging model

CD11c-EYFP mice [28] were obtained from Dr. Michel Nussenzweig, The Rockefeller University, New York, NY; hCD2-DsRed mice [29] were from Dr. Dimitris Kioussis, The National Institute for Medical Research, Mill Hill, London, U.K. The mouse strains were interbred to yield a double reporter strain. Male and female mice 6 to 12 weeks of age were used for experiments, and they were euthanized by CO_2_ inhalation followed by cervical dislocation. The B-ALL mCer cell line was generated by transducing fetal liver cells with a BCR-Abl p190 construct, following by transduction with a plasmid encoding the mCerulean fluorescent protein [30]. These and other cells were cultured in IMDM medium containing 10–20% fetal bovine serum, 1% β-mercaptoethanol, and 1% penicillin/streptomycin. Mice were injected with 1.25 × 10^5^ B-ALL cells i.v. in 200 μL Hank’s Balanced Salt Solution (HBSS). At specified time points, mice were anesthetized using a 10 mg/ml ketamine and 1 mg/ml xylazine cocktail i.p. (at a dose of 10 μl/g of body weight), followed by an injection of 50 μl every 20–30 min to maintain anesthesia. After determining complete anesthesia by toe pinch, the scalp skin and membrane beneath were removed surgically to access the skull bone marrow for imaging. Following this procedure, mice were head-immobilized with a custom-made stereotactic holder on a heated microscope stage in enclosure maintained at 37 °C throughout the entire imaging session.

#### Lung metastases imaging model

Cyan fluorescent MCA-205 fibrosarcoma cells were generated by transducing the parental cell line with mCerulean vector. The reporter mice, as described for the leukemia model, were injected with 2.5 × 10^5^ MCA-205-mCer cells in 200 μL HBSS through the tail vein. At specified time points, mice were anesthetized as for the leukemia model, and were tracheotomized and intubated for mechanical ventilation (Inspira, Warner Instruments). The large lung lobe was exposed by partial ribcage excision and immobilized for imaging using a heated suction holder system (STH-2, VueBio.com).

#### Intravital oxygen and dynamic cell imaging by FaST-PLIM

In order to visualize oxygen tensions in vivo, we injected mice with 40 μl of 1.7 mM solution of PtP-C343 oxygen probe [19]. Imaging was performed using a two-photon microscope system consisting of two titanium sapphire femtosecond lasers (Mai Tai, Spectra Physic), two electro-optical modulators (EOM) (Linos), polarization-based merge optics, SP5 laser scanner, DMI6000 microscope chassis (Leica Microsystems), and 25x NA 1.1 water immersion objective (Nikon) (Additional file 1: Figure S1). The emitted photons were collected by a four-channel non-descanned detector (Leica) consisting of two hybrid photodiode photomultiplier detectors (HyD) and two photomultiplier detectors optically arranged with appropriate dichroic mirrors and bandpass filters (Semrock). Laser gating and time correlated single photon counting were managed by the SPC-150/DP-120 subsystem (Becker & Hickl). For the *concurrent* mode of FaST-PLIM imaging, one laser was used at 875 nm and ~ 1.4 W infrared power at laser output, and the beam intensity was decreased down to 12.5 or 25% by a neutral density filter and passed through one EOM and dual stacked broadband polarizers. At these settings, infrared power measured at the objective back aperture was 25–55 mW. In *concurrent* mode, images were captured in 256 × 256 format (2.42 μm pixel size) with bi-directional scanning. Pixel dwell time of ~ 200 μs was obtained at 5 Hz line scan frequency, or 100 μs at 10 Hz, for example. During each pixel dwell time, the EOM/SPC150-modulated laser beam was off for 10 μs, then pulsed on for 10–30 (typically 20) μs, and again off for the remaining time. The pulse duration was adjusted for the best balance of fluorescence and phosphorescence brightness. Signals from one HyD detector were routed by B&H multi-channel scaler to the “phosphorescence” channel during the laser-off times, and to “fluorescence” channel during laser-on times. Simultaneously, entire signals from all four detectors were collected by the Leica SP5 system.

The rate of oxygen imaging in FaST-PLIM, i.e., the number of accumulated phosphorescence frames needed for given precision of lifetime estimations, was determined largely by the rate of phosphorescence photons and the noise. To minimize the number of frame accumulations, we considered the trade-off between temporal vs. spatial resolution. Accepting spatial resolution in the order of a single lymphocyte’s diameter (~ 10–20 μm), we were not concerned with limiting laser power to remain within the quadratic range (which would be required for a diffraction-limited excitation spot size) [24], and we applied short-radius circular pixel binning prior to automated decay curve fitting for each pixel position. This way, and using PtP-C343 at biocompatible concentrations, enough photons were accumulated to generate one lifetime image in 1–5 min, i.e. in just 3–12 scans. We used *concurrent* phosphorescence and fluorescence acquisition mode for specimens whose fluorescence was sufficiently bright when excited at wavelengths appropriate for excitation of PtP-C343. If one of the fluorescent labels was dim or required excitation at a different wavelength, we performed acquisition in *sequential* mode, in which cellular motility was imaged using dual laser excitation at the full duty cycle immediately before or after (or between) recording the phosphorescence. For *sequential* FaST-PLIM imaging, the fluorescence component data were captured for 5–30 min in 512 × 512 format (1.41 μm pixel size) at 600 Hz bi-directional line scan frequency, in up to eight channels (two lasers x four detectors), followed (or preceded) by the phosphorescence component for 1–5 min, as in the concurrent mode.

#### Lifetime data analysis

Post-acquisition image processing pipeline included conversion of lifetimes into oxygen partial pressures (pO_2_) and co-registering the phosphorescence channel with fluorescence channels for oxygen and cell movement cross-examination. Phosphorescence lifetimes were determined using single-exponent model in SPCI 6 software (B&H):

$$ {y}_n={A}_n{e}^{-\left(t-{t}_n\right)/{\tau}_n}+{b}_n $$

Where *y* is the number of photons, assuming analog response and no shot noise; *n* is the pixel index, *A* is the amplitude; *t* is time; *t*_*n*_ is the decay start time; τ is the lifetime; and *b* is the background (offset). The offset setting was for the pre-pulse time bins. Up to ~ 5 circular binning was used to reach the average amplitude of at least 100 photons per binned pixel, with less binning for stronger signals. At 5 binning, the effective spatial resolution of phosphorescence was in the order of ~ 25 μm, i.e., comparable to the diameter of a T cell. In incomplete decays with non-descanned detection, decay carryover will cause overestimation of the offset, which would propagate to lifetime. At the current conditions, there is maximum 0.3% offset error (for τ = 50 μs, pulse-to-pulse time ≥ 290 μs), lifetime error similar. The phosphorescence lifetime images were converted into pO_2_ (mmHg) images based on a Stern-Volmer calibration curve [19] (Microsoft Excel 2017).

#### Contextual cell tracking

The fluorescence lif-format files and pO_2_ image sequences were combined in the Imaris 8.4.2 analysis software (Bitplane AG, Saint Paul, MN). Voxel dimensions were specified according to the objective used for image acquisition. If drift was present, it was corrected based on tracked landmark features. T cell motility was analyzed using the automated spot detection followed by autoregressive spot tracking and manual error correction. Quantitative analyses were done on all tracks with duration > 5 min, typically. Oxygen tension channel was Gaussian kernel 3 smoothed and the individual T cell-centered local oxygen tensions were the averages in cell-sized circular areas (~ 8 μm diameter).

#### Software for acquisition and analysis

Image acquisition and initial processing was performed using LAS 2.7 (Leica Microsystems) for intensity data, and SPCM 9.77 (Becker & Hickl) for time-resolved data. Further image processing and analysis was conducted using SPCImage 6 and 7 (B&H), IMARIS 8.4.2 (Bitplane, Inc.), ImageJ 1.51w (NIH), Anaconda Python Distribution 5.1 and Python 3.5.5.

#### In silico simulations

Simulations were performed using Microsoft Excel 2016 with Analysis Pack, Virtual Basic for Applications (VBA) and Realstats Resource Pack (real-statistics.com). Simulation conditions were: 150 photons per decay, 3:1 phosphorescence to background ratio, Monte Carlo Poisson (shot) photon noise, 256 time bins, 10 μs pre-pulse or no pre-pulse, 20 μs excitation gate. For the “conventional” complete decay method, the dwell time was 500 μs, and for the partial decay with or without pre-pulse, the dwell time was 100 μs.

#### Statistical analysis

The two-sided student’s t-test was used to evaluate the null hypothesis that there was no difference between two groups, and a *p*-value of < 0.05 was considered statistically significant a priori. ANOVA with Tukey multiple comparison test was used where the means of more than two groups were to be compared simultaneously. Pearson’s correlation was used to evaluate correlations between two variables. r values > 0.1 with corresponding *p* values < 0.05 were considered to be significant a priori.

## Results

### Performance of FaST-PLIM in oxygen imaging in vitro and in vivo

To evaluate FaST-PLIM, we imaged PtP-C343 solutions that were equilibrated with air (21% oxygen) or deoxygenated using glucose/glucose oxidase/catalase enzymatic system. We observed the expected short and long lifetimes, respectively (Additional file 1: Figure S3A). The deviation of lifetime values across the fields of view was satisfactorily narrow (respectively ~ 10 and 12% full width at half maximum). Next, we considered that although using incomplete decays would not affect lifetime estimation for monoexponential decays, some underestimation could occur for a probe with multiple lifetime components such as PtP-C343 [16, 31]. Tested experimentally, the minimum duration of a decay measurement before the apparent lifetime of PtP-C343 departed from that measured by complete decay could be as short as 70 μs (Additional file 1: Figure S3B). Independent measurements confirmed that the oxygen calibration plots constructed using complete decays (500 μs acquisition) and shortened decays (70 μs) were nearly super-imposable for PtP-C343 (data not shown). Thus, with the duration of decay measurement typically set at 170 μs for a 200 μs pixel dwell time, the precision of pO_2_ measurements by FaST-PLIM was unaffected by the probe’s multiexponential decay characteristic.

To further test the oxygen imaging system in vivo, we infused PtP-C343 into C67BL/6 mice and, under anesthesia, imaged the lung while the mouse was mechanically ventilated with air (~ 21% oxygen), and then 100% oxygen, and then returning the breathing back to the air. We observed an expected decrease in the phosphorescence lifetimes across the lung interstitial tissue upon inhalation of 100% oxygen, and then an increase back to the baseline following transition back to air (Fig. 1d). For an opposite response, we imaged PtP-C343 phosphorescence lifetimes in the calvarial bone marrow (BM). Imaging through the intact skull, we observed phosphorescence in the lumens of blood vessels and, consistent with permeability of BM vasculature, in interstitial space at lower intensities (Fig. 1e). Lifetime images revealed expected gradients of lifetime values with increasing lifetimes along the length of capillaries with the direction of blood flow (Fig. 1e and f). The calculated average BM pO_2_ of ~ 27 mmHg was in agreement with a prior report (< 32 mmHg) [19]. As expected, we observed a widespread increase of the phosphorescence lifetimes within minutes of specimen asphyxiation (Fig. 1g). A more detailed analysis of oxygen gradients in healthy and pathological tissues is a topic of separate report. Thus far, these in vitro and in vivo results demonstrated the capacity of FaST-PLIM to image the entire range of pO_2_ at a pixel binning-dependent spatial resolution of about 25 μm in one to several minutes.

### Oxygen experience and motility dynamics of T cells in healthy vs. leukemic bone marrow

Using FaST-PLIM, we investigated the motility of BM infiltrating T cells in the context of available oxygen. For this purpose, we infused PtP-C343 and, in some experiments, TRITC-dextran (a blood supply label) intravenously into healthy dual fluorescent mice harboring T cell lineage reporter hCD2-DsRed [29] and dendritic cell lineage reporter CD11c-YFP [28] transgenes. Using the concurrent acquisition with the scanning rate of one frame in 26 s, we captured the pO_2_ along with fluorescence (Fig. 1h). In these specimens with additional strong fluorescence, we noted that the use of pre-pulse in our FaST-PLIM method was very effective in reducing the undesirable lifetime/offset cross-talk resulting from residual fluorescence excitation during the ‘laser-off’ period due to imperfect dynamic range of the EOM laser gating device (Additional file 1: Figure S4). As expected, the frame rate of one multi-channel fluorescence image in 26 s was suitable for tracking relatively fast moving cells such as T-lymphocytes. Combining T cell tracks with the pO_2_ images revealed individual T cell movements in the context of local pO_2_ landscapes (Fig. 1h-i). The observed range of T cell pO_2_ and instantaneous velocity values spanned from, respectively, less that 5 to about 60 mmHg and 0 to several tens of μm/s and the most frequently observed T cell pO_2_ was in the 20–30 mmHg range (Fig. 1j). Using the sequential FaST-PLIM mode, we tracked T cells either before or after the phosphorescence image capture, and verified that T cell migration velocities were unaffected by oxygen imaging, and vice versa, that oxygen levels were similar before and after cell tracking session. The concurrent and sequential FaST-PLIM modes were equivalent in revealing T cell motility and oxygen distribution (data not shown).

**Fig. 2:**
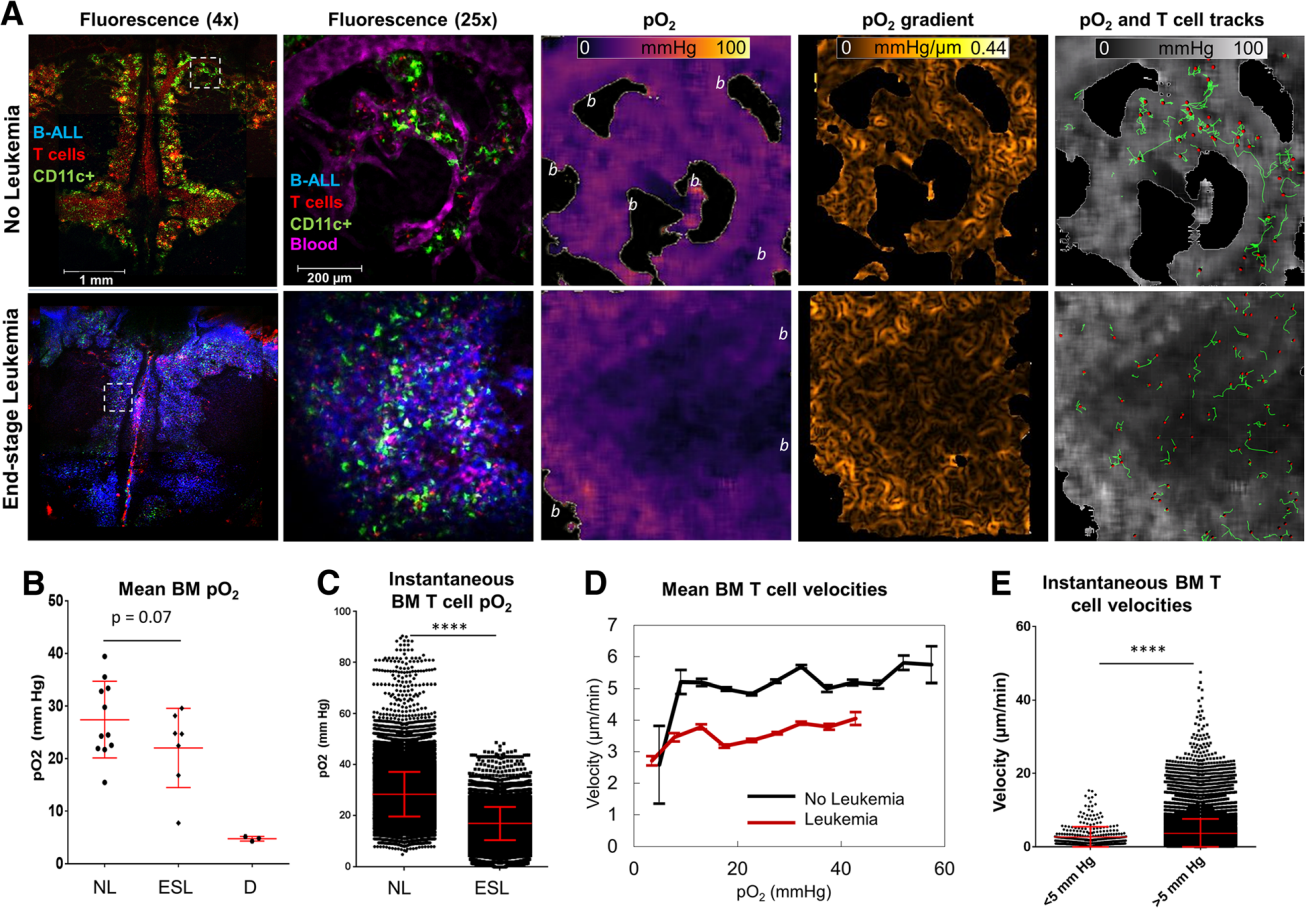
Relationship between T cell dynamics and oxygen distribution in bone marrow of mice with or without B-ALL. **a** Low magnification confocal images and high magnification FaST-PLIM images of the calvaria. FaST-PLIM images are for the fields of view indicated with the dashed squares. “*b”* indicates the areas of bone and no signal from the probe. The specimens are exposed skulls in anesthetized mice transgenic for CD2-DsRed (red, T cells) / CD11c-YFP (green, myeloid cells) and infused with blood tracer dye dextran-TRITC and PtP-C343 probe. The blue represents B-ALL-mCerulean fluorescence in the mouse inoculated, about 2 weeks earlier, with the leukemia. **b** Mean oxygen tension in the entire BM cavity of non-leukemic (“NL”), end-stage leukemic (“ESL”), and asphyxiated (“D”) mice. Each symbol represents a single FOV. **c** Oxygen experience of bone marrow T cells. Each point represents a readout for one T cell in one time point (*n* > 30,000 per group). **d** Overall relation between the average T cell velocity and pO_2_. The range was analyzed in intervals of 5 mmHg. **e** Comparison of T cell velocities in pO_2_ below or above 5 mmHg in leukemic mice. Each symbol represents a T cell instance (n > 30,000 per group). Pooled from more than 2 FOVs per mouse in at least 3 mice per group. Symbol * denotes *p* < 0.05 determined by Student’s T-test. Error bars show standard deviations

Development of low oxygen partial pressures in leukemic bone marrow is a known characteristic of acute leukemias including in patients [4, 32–34]. To compare tissue oxygenation and T cell dynamics in healthy BM to that in BM with acute lymphoblastic leukemia (ALL), we infused the immune competent CD2-DsRed/CD11c-YFP dual fluorescent mice with syngeneic cyan-fluorescent leukemia cells transformed by the BCR-Abl fusion protein expression. In this B-cell ALL model, cancerous cells invaded the BM and caused animal demise within several weeks. Focusing on advanced-stage disease, oxygen images revealed frequent presence of pockets of considerable hypoxia (less than 5 mmHg of oxygen) that were interspersed between perivascular regions of relatively high oxygen and spanned tens to hundreds of micrometers each (Fig. 2a). We observed lower average oxygen tensions (pO_2_ = 22.0 mmHg vs. 27.4 mmHg) over whole fields of view, and slightly shallower oxygen gradients in the leukemic BM compared to that in healthy mice, but variability was large and the differences in whole field of view pO_2_ values did not reach statistical significance (Fig. 2b and Additional file 1: Figure S5A). Remarkably, T cells were present in leukemic BM, including in advanced-stage ALL (Fig. 2a). The local oxygen levels experienced by individual T cells in leukemic BM were quite low, averaging only ~ 18 mmHg, and were substantially lower than that experienced by T cells in healthy BM (~ 29 mmHg) (Fig. 2c). This relationship was related to an increased proportion of T cells experiencing low (< 10 mmHg) and very low (< 5 mmHg) levels of oxygen in leukemia (Additional file 1: Figure S5B).

Time lapse recordings revealed overall slower pace of T cell migration in leukemic BM compared to that in healthy BM (respectively, 3.6 μm/min vs. 5.2 μm/min, *p* < 0.05). Using FaST-PLIM, we performed contextual analysis of the instantaneous measures of T cell dynamics with respect to the local pO_2_ for each cell position in its path of movement. Both in healthy and leukemic BM, T cell velocity in sites of pO_2_ below 5 mmHg was slower than in sites of pO_2_ above this level. This analysis revealed a coincidence between low T cell velocities and oxygen levels below 5 mmHg, i.e., in areas of hypoxia (Fig. 2d). In the hypoxic areas, T cell velocities averaged only 2.70 μm/min compared to substantially higher velocities above 5 mmHg of oxygen (Fig. 2e). A similar relationship was found for T cell acceleration, which was lower in pO_2_ below 5 mmHg compared to that in higher levels (Additional file 1: Figure S5C). Consistent with slower migratory velocities in areas of hypoxia, the proportion of T cell time spent in the areas with pO_2_ below 5 mmHg was much higher in BM with advanced-stage ALL than in the controls (1.07% vs. 0.007%, respectively). These results showed that T cells present in the leukemic BM were exposed to the hypoxic microenvironments and their motility was compromised in the leukemic BM and especially in the sites of tissue hypoxia.

### Decreased motility of TILs in solid tumor core hypoxia and re-invigoration by hyperoxygenation

**Fig. 3:**
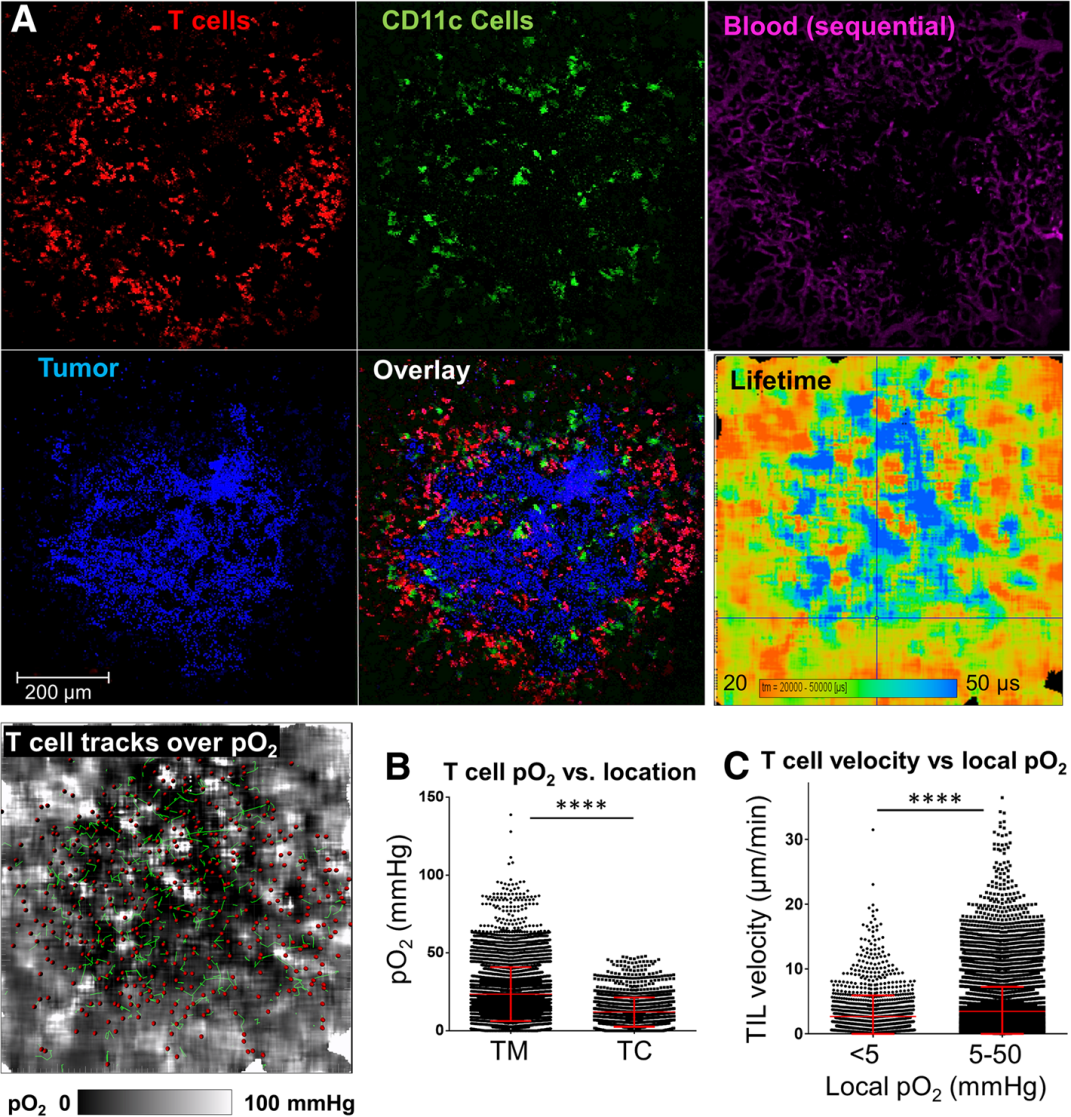
Oxygen distribution and T cell dynamics in solid MCA tumors in lungs, and T cell response to supplementary oxygen. **a** Intravital FaST-PLIM images of solid tumor in the lung of a mouse transgenic for hCD2-DsRed (T cell reporter, red) and CD11c-YFP (dendritic cell/macrophage reporter, green). Cancer cells (MCA-205) express mCerulean fluorescence (blue). Blood supply was labeled with TRITC-dextran (purple), co-infused with PtP-C343. T cells were tracked for 30 min and shown is one time point. The lifetime is that of PtP-C343 phosphorescence, contemporaneous with the corresponding fluorescence images. **b** pO_2_ experienced by T cells in the tumor margin (TM) vs. tumor core (TC) during the mouse breathing air. **c** T cell instantaneous velocities in the local pO_2_ < 5 mmHg vs. that in pO_2_ > 5 mmHg. Each symbol represents a T cell instance (*n* > 5000). Graphs display data from representative tumor FOVs. * denotes *p* < 0.05 determined by Student’s T-test. Vertical bars represent standard deviations

**Fig. 4:**
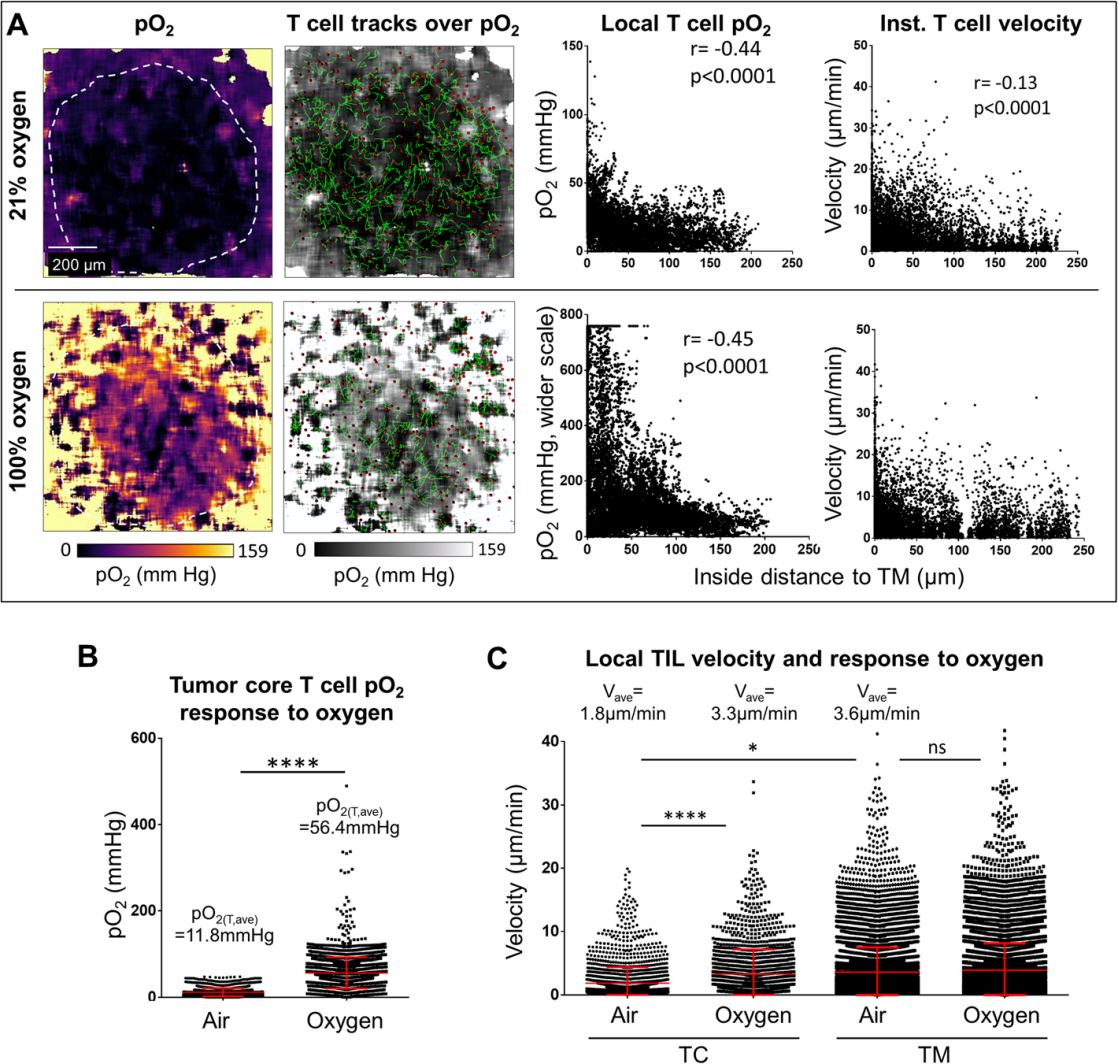
Response of tumor infiltrating T cell motility to changing the specimen breathing from air (21% oxygen) to 100% oxygen. **a** The same tumor as in Fig. 3 was imaged by FaST-PLIM while the mouse was breathing air (upper row) or about 30 min after breathing change to oxygen (lower row). From the left: images of oxygen tensions (pO_2_), T cell tracks overlaid on pO_2_; relation of intratumoral T cell pO_2_ experience to the distance to tumor margin; corresponding relation of T cell instantaneous velocity to the distance to tumor margin. **b** Local pO_2_ for T cells located in tumor core when the mouse was breathing air vs. that for breathing oxygen. **c** Local velocities of tumor infiltrating T cells (TIL) in tumor core (TC) or tumor margin (TM) while the mouse was breathing air and after breathing oxygen. Each symbol represents a T cell instance (n > 5000). Graphs display data from representative tumor FOV. * denotes p < 0.05 determined by Student’s T-test. Vertical bars represent standard deviations. r = Pearson’s coefficient

Decreased oxygen availability is a typical characteristic of solid tumors. To analyze T cell-oxygen interactions in solid tumors, we infused the dual reporter hCD2-DsRed / CD11c-YFP mice with MCA205-mCer fibrosarcoma cells through the tail vein, which resulted, two weeks later, in multiple tumor nodules in the lung. These tumors were infiltrated by T cells, and most of the TILs were distributed around the tumor periphery, while the rest (~ 10–30%) penetrated the tumor itself (Fig. 3a). Oxygen images showed that most of the large lung tumor nodules exhibited prominent hypoxic areas (pO_2_ below 5 mmHg) in tumor cores, and that the hypoxic regions were interspersed with areas of shallow normoxia near blood vessels (Fig. 3a). The average T cell in the tumor core experienced only 11.8 mmHg oxygen vs. 23.3 mmHg in the margin (*p* < 0.05) (Fig. 3b). Reminiscent of the bone marrow model, the contextual analysis of the relation between TIL motility and the local oxygen concentrations in solid tumors showed that TILs exhibited slower migration kinetics in the sites of hypoxic oxygen tensions (i.e., below 5 mmHg) than in the sites where the local pO_2_ was greater than 5 mmHg (Fig. 3c). Overall, when the animal was ventilated with air (~ 21% oxygen), we observed an inverse correlation between T cell motility inside tumor and the T cell distance from tumor margin (r = − 0.13, *p* < 0.0001), which correlation was similar to that between T cell oxygen experience and the T cell to tumor margin distance (Fig. 4a). In fact, many of the tumor core T cells appeared stalled.

Using this model, we tested TIL response to animal hyperoxygenation. For this purpose, we changed the animal ventilation from air to 100% oxygen and re-assessed both the local oxygen levels and TIL motility by FaST-PLIM. Within minutes of supplementary oxygen delivery, oxygen levels increased throughout lung tissue including the hypoxic tumor cores, which became partially re-oxygenated (Fig. 4a and b). On average, the oxygen experienced by T cells within the entire tumor microenvironment increased from 21.7 mmHg to 180 mmHg, and in the tumor core from 11.8 mmHg to 56.4 mmHg. Interestingly, whereas the average T cell velocity was very slow in the tumor core during air breathing (1.84 μm/min), which was only about half of that in the margins at the same time (3.62 μm/min, p < 0.05) (Fig. 4a and c), many of the stalled tumor core T cells became motile within minutes of tumor hyper-oxygenation. On average, the velocities of the T cells in tumor core increased to 3.30 μm/min on average (p < 0.05), i.e., close to the velocities of T cells in tumor margins, in response to animal hyperoxygenation. At the same time, T cell velocities in the tumor margin remained largely unchanged (Fig. 4c).

## Discussion

Prior studies demonstrated that animal hyperoxygenation may enhance the efficacy of anti-tumor immune therapies [9, 35]. In this report, we used a novel imaging regimen that enables high-resolution oxygen imaging in compatibility with fluorescence-based cellular tracking to reveal the in vivo relationship between the local oxygen partial pressures in tumors and the motility dynamics of tumor infiltrating T cells. The ability of our FaST-PLIM method (Fast Scanning Two Photon Phosphorescence Lifetime Imaging Microscopy) to map in vivo oxygen gradients along with cellular dynamics revealed that tumor hypoxia contributes to TIL motility slowdown. Our findings of hypoxia in the core regions of solid tumors as well as in the bone marrow of mice with late-stage leukemia are consistent with previous reports using other methods of oxygen evaluation [4, 36]. However, our determination that TILs were less agile at oxygen concentrations below 5 mmHg, and that stalled T cells inside solid tumors could be re-invigorated by tumor hyper-oxygenation, represent novel findings.

Cellular motility is associated with high energy expenditures to generate ATP supply to power the activity of molecular motors and other cellular processes involved in cell movements. Activated lymphocytes meet their energy demands largely through oxygen-independent metabolism such as glycolysis and glutaminolysis, compared with primarily oxidative metabolism in naïve and memory states. This metabolic shift is thought to be advantageous for lymphocyte functioning in the sites of hypoxia-causing pathologies including in cancer [37–43]. In this respect, the previously reported effectiveness of hyperoxic treatment in the improvement of anti-tumor immune responses [9, 35] and, here, our finding of oxygen-effected TIL motility reinvigoration, together suggest that this metabolic shift is not absolute, and that oxygen is still utilized by TILs or TIL subsets such as for cellular migration. Considering that the increase of TIL motility in response to hypoxia reduction was relatively rapid (minutes), our results suggest that the reliance of TIL motility on oxygen reflects an intrinsic metabolic process. Given our results in the leukemia model showing T cell motility decrease in advanced disease across oxygen levels above 5 mmHg (Fig. 2d), hypoxia cannot be the only factor behind the slowdown of T cells in leukemic marrow. Unfortunately, the extent of hypoxia contribution to this slowdown could not be determined because supplementary oxygen did not significantly increase bone marrow pO_2_ during imaging, likely reflecting a high metabolic activity and vascular disorganization in this model (Harutyunyan, Rytelewski et al., manuscript in preparation). Thus, we do not exclude the possibility of additional mechanisms, such as through modulation of other cells and/or the microenvironment.

While animal hyperoxygenation restored the motility of T cells in a tumor’s core in apparent correlation with alleviating the local hypoxia (defined as pO_2_ < 5 mmHg), this treatment did not substantially change the motility of T cells in the tumor margin. Our favored explanation for this observation is that T cell motility is limited by oxygen availability only in quite low oxygen levels; i.e. below about 5–10 mmHg, whereas tumor margins were relatively well oxygenated and T cell motility there was already quite high. This oxygen dependence pattern is consistent with the highly non-linear nature of the dependence of the cellular energy state (ATP/[ADP*Pi]) on the average pO_2_ [44] and references therein. Our results indicate that there is a certain threshold in oxygen concentration, below which the energy state is insufficient for the cellular machinery involved in motility to operate at its normal level. On the other hand, our results suggest that oxygen is no longer a limiting factor for TIL motility once its levels are greater than this threshold, currently estimated at about 5 mmHg, in keeping with the capacity of activated T cells for non-oxidative metabolism. FaST-PLIM should be instrumental for further mechanistic elucidation of these and other relationships, including in the context of therapeutically-induced responses. In combination with appropriate cellular lineage and functional reporters, it should also reveal other oxygen dependencies of various immune cell types and lymphocyte subsets and activation stages.

This work introduces the FaST-PLIM method, which represents an easily implemented yet powerful merger of modified two-photon phosphorescence lifetime microscopy with classical two-photon microscopy. The main benefits of FaST-PLIM include the improvement of imaging efficiency for lifetime imaging and compatibility with fluorescence tracing. Its current spatial resolution for oxygen (approximately 5–10 μm in X-Y and 15–20 μm in Z for PtP-C343) is suitable for visualizing oxygen gradients and for reading out pO_2_ in a location of a single cell. The corresponding temporal resolution of one to several minutes for oxygen is appropriate for biological systems where oxygen gradients are steady or slowly changing. The response to oxygen inhalation is one example. On the fluorescence side, the attained rate of scanning affords the ability to track the dynamics of cellular processes such as that of fast T cell movements. Because the corresponding data can be acquired concurrently, the relation between cellular dynamics and oxygen mapping is relatively robust when faced with slow oxygen changes. The alternative, *sequential* FaST-PLIM mode enables capturing of dim fluorescence at an unrestrained sensitivity at the price of a temporal shift between the oxygen and fluorescence recordings.

Significant performance improvement in oxygen imaging using FaST-PLIM was realized by measuring ‘incomplete decays’ in conjunction with the use of pre-pulse. The high temporal efficiency of the FaST-PLIM strategy comes in part from its effective use of the system’s pixel-to-pixel lag time, which lag time is otherwise unproductive and may be difficult to avoid. The use of incomplete decay acquisition approach is potentially complicated by the presence of multiple lifetime components in probe phosphorescence when using single exponential fitting [31]. Due to the structural complexity and a multitude of conformations constituting the excited state ensemble at any given time, phosphorescence decays of dendritically-protected phosphorescent probes, such as PtP-C343, are characterized by multi-component decays [16], and the relative amplitudes of short and long lifetimes could, in principle, vary over the pO_2_ range. These phenomena have been addressed by us (SV) in detail previously [16, 26]. In short, non-single-exponentiality, if treated properly, does not reduce accuracy of the lifetime/pO_2_ determination. Indeed, as long as the decay model used to fit the experimental data is the same as that used in the calibrations, Stern-Volmer like plots can be applied to convert the ‘apparent’ lifetimes into pO_2_ even if the decays are not fitted with the best possible χ^2^ values. Thus, considering that achieving high pO_2_ accuracy is more reliant on the mathematical uniformity between measurements and calibration methods than on the fitting model exactness, we opted for single exponential fitting. Having said that, the issue of the decay exponentiality was addressed by us experimentally (Additional file 1: Figure S3B and C) and by mathematical modeling in this work as well. Our results show that the single exponent model is completely adequate for fitting phosphorescence decays truncated at no less than 70 μs, as was done in our experiments. Furthermore, comparison of the Stern-Volmer plots obtained using such truncated decays and of those constructed using ‘complete’ decays revealed that the difference between the two was negligible. Hence, we concluded that single exponent model, combined with our decay truncation approach, is adequate, and it should remain adequate down to the single cell resolution level, as long as the focal volume contains an ensemble of excited state molecules. This was certainly the case in our experiments. Another potential source of oxygen measurement error is related to the fact that the pre-pulse readout consists of both the true offset and superimposed tail photons from the previous pixel. If the carryover contribution is significant and unaccounted for, this effect of non-descanned detection may in principle lead to lifetime underestimation. However, the impact of signal carryover is relatively benign at excitation intervals of ~ 4x the longest lifetimes [31], which is our current implementation. In particular, the combined carryover is minimized to as little as < 1% of the phosphorescence amplitude for decay time of 50 μs and even less for shorter lifetimes, and no lifetime underestimation was observed for incomplete decays of more than 70 μs. Overall, the precision of lifetime and pO_2_ estimations in FaST-PLIM was determined largely by the noise, hence by the number of accumulated phosphorescence frames.

The FaST-PLIM method could be pushed toward higher temporal performance for both oxygen and fluorescence imaging frame rates, for example to study oxygen landscape fluctuations and/or faster cellular responses. This could be accomplished in several ways, including by further shortening of pixel dwell time and by sacrificing some precision of pO_2_ measurements. For pixel dwell time shortening, possible approaches include to use a more involved fitting model that would account for the effect of pixel-to-pixel decay tail carryover, and to calibrate the probe for short decay measurements. Of note, because shortening of unitary acquisition cycles is contingent on using the frame-by-frame imaging mode for avoiding triplet saturation, FaST-PLIM is not advantageous for solitary spot measurements with high temporal resolution per pixel. Nonetheless, FaST-PLIM is potentially beneficial for multi-spot measurements, where it could accelerate data acquisition by enabling incomplete decay measurements, if the ability to move the excitation rapidly between the spots is available. The spatial resolution of FaST-PLIM could be increased as well, up to diffraction limit, by using less binning, denser pixel spacing, and lower laser powers; however, requiring more frame accumulations, the speed of such imaging would be proportionally slower.

## Conclusions

By directly visualizing the relationship between local oxygen concentrations and TIL motility using FaST-PLIM, this study reveals that tumor hypoxia plays a quantitative and reversible role in TIL migratory slowdown, and it demonstrates that the slow immune surveillance motility of tumor core TILs can be boosted by alleviating tumor hypoxia through breathing supplementary oxygen. These results add to the variety of mechanisms by which tumor hypoxia disrupts tumor immune surveillance. Considering that non-motile T cells cannot interact with enough target cells for their cytotoxic functions to be consequential [45, 46], our results bring up the possibility that the previously reported therapeutic benefits of supplementary oxygen in experimental tumor immunotherapy could be in part mediated by TIL motility upregulation. Exactly how the role of oxygen availability TIL motility is mediated at a molecular level, and how the enhancement of TIL motility by tumor oxygenation would contribute to the efficacy of tumor immune therapies remains to be uncovered, for which task the FaST-PLIM method is advantageous. With the ability to map tissue oxygen in vivo at cellular resolution along with cell dynamics, the FaST-PLIM method is useful for mechanistic studies in a variety of living biological systems for which oxygen is of interest, ranging from body morphogenesis to stem cell mobility, tissue healing, tumor invasiveness and, as we studied, tumor immune surveillance.

## Additional file

Additional file 1: **Figure S1.** Schematic diagram of signal routing for FaST-PLIM. **Figure S2.** Performance and precision benefits of Pre-Pulse in FaST-PLIM. **Figure S3.** Characterization of FaST-PLIM performance in vitro. **Figure S4.** Reduction of lifetime-offset cross-talk using Pre-Pulse. **Figure S5.** Additional quantification of oxygenation and T cell dynamics in healthy and leukemic BM by FaST-PLIM. **Figure S6.** Example of an analysis of T cell motility and oxygen tensions with respect to the distance from tumor inside to tumor margin. (PDF 1667 kb)

## Abbreviations

2p: Two-photon
ALL: Acute lymphoblastic (lymphocytic) leukemia
EOM: Electro-optical modulator device
FaST-PLIM: Fast-scanning two-photon phosphorescence lifetime imaging microscopy
PLIM: Phosphorescence lifetime imaging microscopy
pO_2_: Partial pressure of oxygen
TIL: Tumor-infiltrating lymphocyte
