# Supplementary material for: Merger of dynamic two-photon and phosphorescence lifetime microscopy reveals dependence of lymphocyte motility on oxygen in solid and hematological tumors
Source: J Immunother Cancer. 2019 Mar 18;7:78. doi: 10.1186/s40425-019-0543-y (PMC6423744; doi:10.1186/s40425-019-0543-y)
Supplement: Supplementary file 1 — Figure S1. Schematic diagram of signal routing for FaST-PLIM. Figure S2. Performance and precision benefits of Pre-Pulse in FaST-PLIM. Figure S3. Characterization of FaST-PLIM performance in vitro. Figure S4. Reduction of lifetime-offset cross-talk using Pre-Pulse. Figure S5. Additional quantification of oxygenation and T cell dynamics in healthy and leukemic BM by FaST-PLIM. Figure S6. Example of an analysis of T cell motility and oxygen tensions with respect to the distance from tumor inside to tumor margin. (PDF 1667 kb) [file 40425_2019_543_MOESM1_ESM.pdf]

Rytelewski et al. ***Merger of dynamic two-photon and phosphorescence lifetime microscopy reveals dependence of lymphocyte motility on oxygen in solid and hematological tumors***

Supplementary Figures

Figure S1. Schematic diagram of signal routing for FaST-PLIM. "fs laser": femtosecond-pulsed infrared lasers. EOM: electro-optical modulator. ND: neutral density filter wheel. P1, P2: stacked broadband polarization cubes. Scanner: fast galvo-mirror scanner. The numbers indicate the central wavelengths and bandwidths of band-pass filters. PMT: photomultiplier. HyD: hybrid detector capable of photon counting.

Figure S2. Performance and precision benefits of Pre-Pulse in FaST-PLIM. Monte Carlo simulation of lifetime measurements by three methods: conventional complete decays (Conv. 500µs), conventional partial decays (without pre-pulse) (Conv. 100µs) and partial decays with pre-pulse (2PreP 100 µs). Respective times refer to pixel dwell times. Simulation parameters: τ = 50 µs, 150 photons per decay, 3:1 phosphorescence amplitude to background ratio, 256 time bins, shot noise, 10 µs pre-pulse duration. **A)** Example photon histograms in a pixel using pre-pulse. Shown are the number 1 and 79 simulated frame scans. **B)** Collection of 20 simulation runs for each method. Lifetime was calculated for every second decay accumulation.

Figure S3. Characterization of FaST-PLIM performance *in vitro*. A) FaST-PLIM imaging of test saline/glucose solutions without (21% oxygen) or with glucose oxidase and catalase (deoxygenated). The graphs are histograms of lifetimes in the corresponding image. **B**) An example of photon counts in one binned pixel from a FaST-PLIM image using a 200 µs pixel dwell time. The green profile represents laser-on gate. The blue dots indicate photon counts and the red line is the fitted function. Using the offset measured during the pre-pulse (0-20 µs), a single exponent model was fitted using the indicated decay time intervals. **C)** The bar graph displays the calculated lifetimes (blue) and the chi-squared fitment quality parameter (red) depending on the duration of decay used for the fitting.

Figure S4. Reduction of lifetime-offset cross-talk using Pre-Pulse. The same image data were analyzed without or with pre-pulse. Resulting lifetimes were plotted vs. the local offset (background) calculated by the model. An undesirable lifetime-offset cross-talk is recognized as the noticeable "trend".

Figure S5. Additional quantification of oxygenation and T cell dynamics in healthy and leukemic BM by FaST-PLIM. **A**) Comparison of mean oxygen gradient steepness in calvaria bone marrow without (NL) or with (ESL) advanced stage acute lymphocytic leukemia. Each dot represents an individual FOV. **B)** Frequency distribution of T cell pO_2 experiences. The dashed lines indicate 5 and 10 mmHg pO_2. **C)** T cell speed accelerations in leukemic BM. Total number of T cell instances >30,000.

Figure S6. Example of an analysis of T cell motility and oxygen tensions with respect to the distance from tumor inside to tumor margin. Lung tumor (MCA205-mCer, blue); overlay with the distance represented as red channel intensity, and overlaid with pO_2 in gray scale, and with T cell tracks.

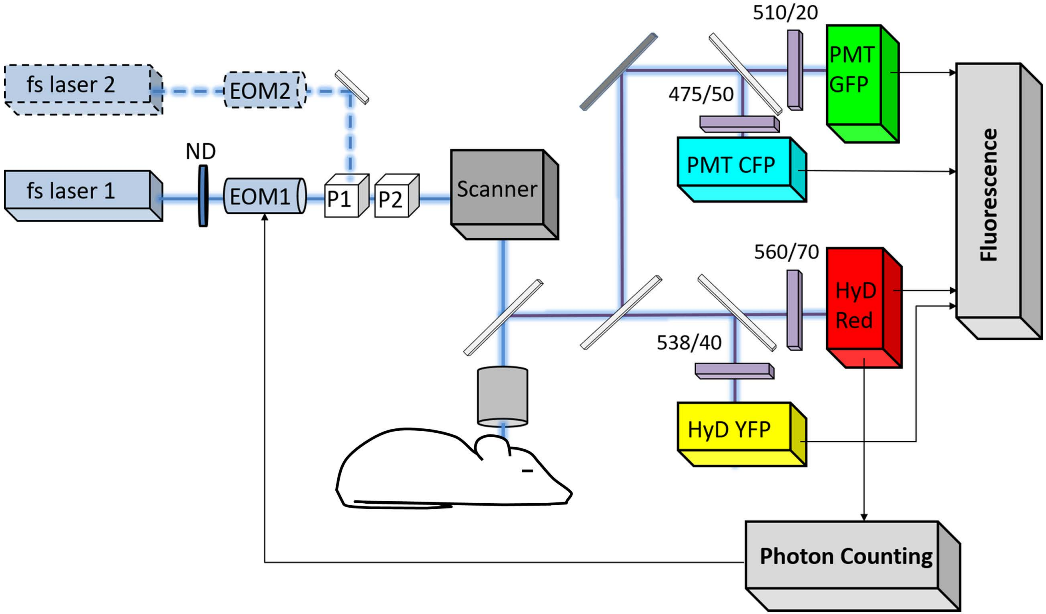

Supplementary Figure 1

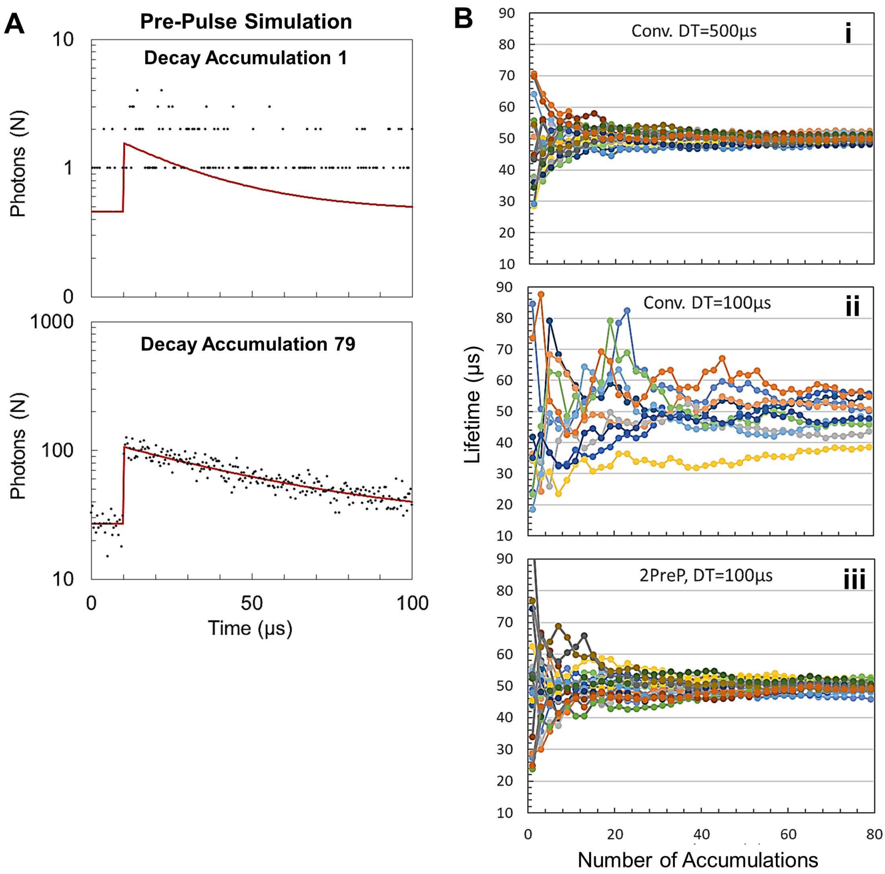

Supplementary Figure 2

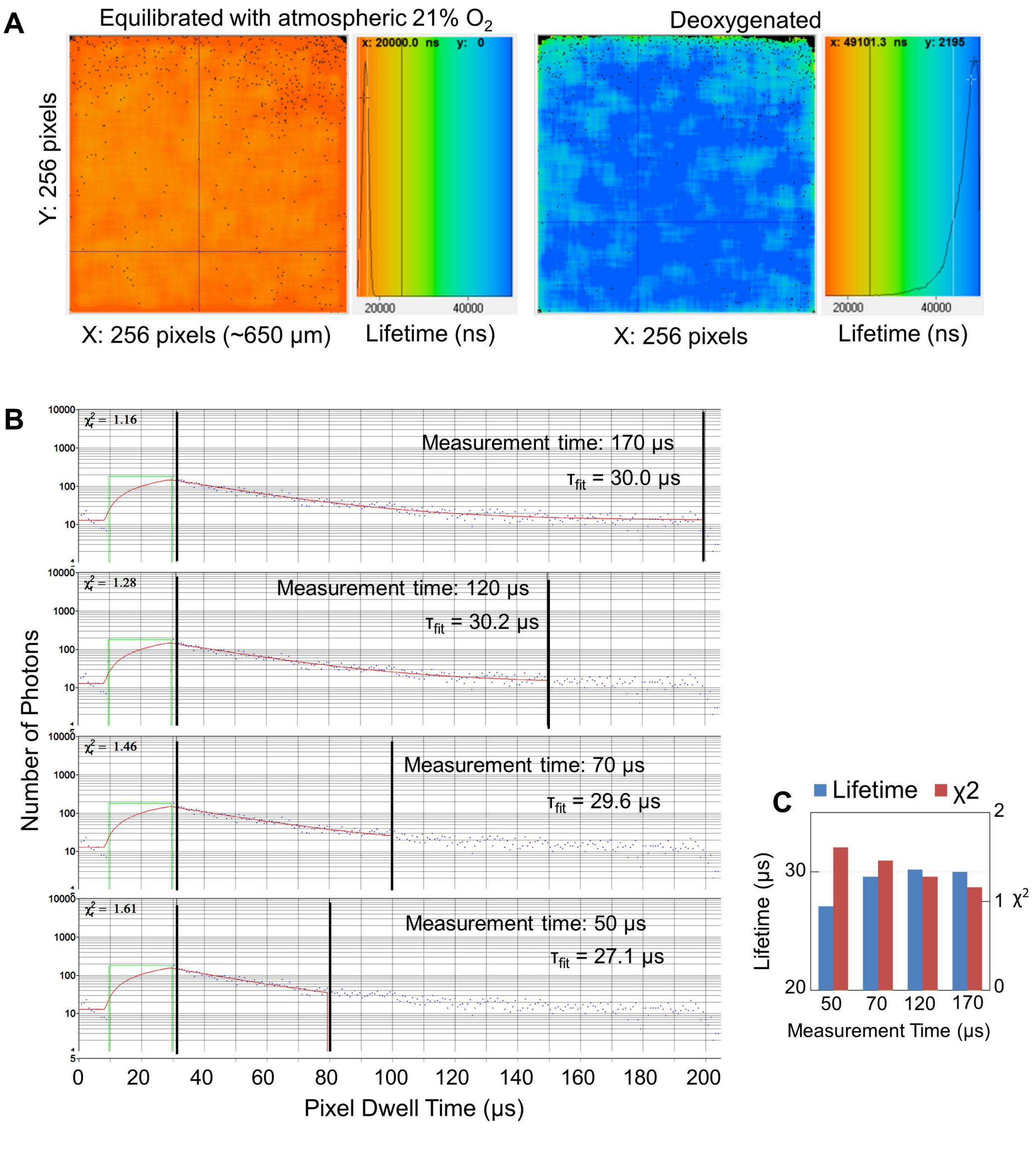

Supplementary Figure 3

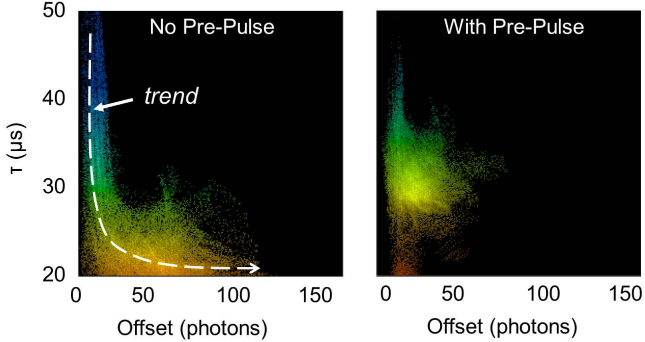

Supplementary Figure 4

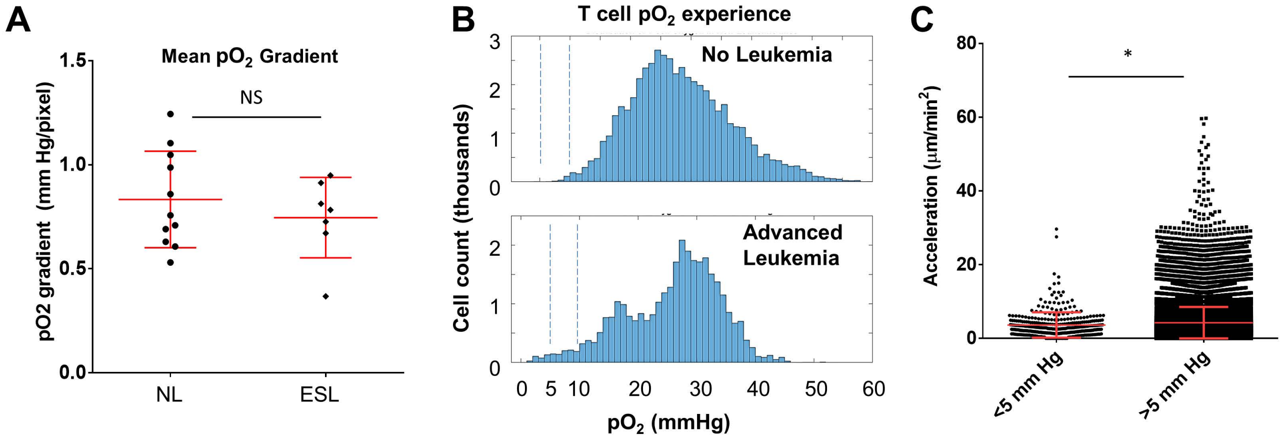

Supplementary Figure 5

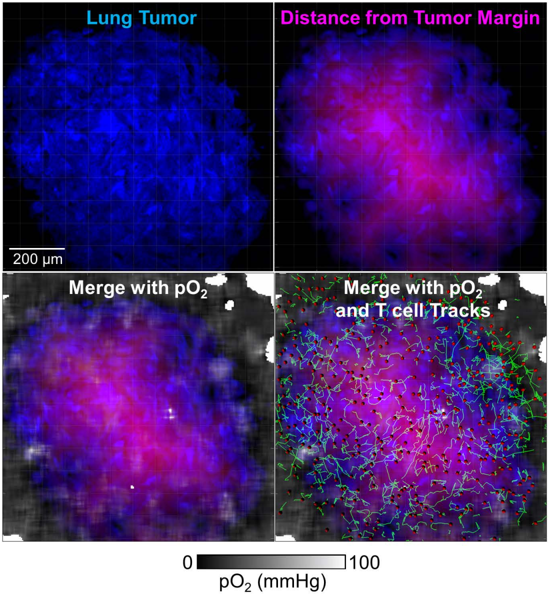

Supplementary Figure 6
